# 

**Published:** 1860-06

**Authors:** 


					﻿No. XIII.	JUNE—1860.	Vol. XV.
NEW YORK
MONTHLY REVIEW
OF
AND
BUFFALO MEDICAL JOURNAL.
EDITED BY AUSTIN FLINT, Jr, M.D,
Prof, of Physiology and Microscopic Anatomy in the New York Medical College.
And J. II. DOUGLAS, M.D.
NEW YORK:
HALL, CLAYTON A CO, PRINTERS,
46 PINE STREET.
Price $3 00 a Year, payable in Advance.
				

